# The behavioral negotiation perspective can reveal how to navigate discord in sustainability transformations constructively

**DOI:** 10.1073/pnas.2414256121

**Published:** 2024-10-31

**Authors:** Johann M. Majer, Simon Columbus, Martin Schweinsberg

**Affiliations:** ^a^Social, Organizational, and Economic Psychology Group, University of Hildesheim, Hildesheim 31141, Germany; ^b^Department of Brain and Cognitive Sciences, Massachusetts Institute of Technology, Cambridge, MA 02139; ^c^Organizational Behavior, European School of Management and Technology, Berlin, Berlin 10178, Germany

Discord and conflict are the main challenges for sustainability transformations. We welcome Patterson, Feola, and Kim’s (PFK) proposed discord view to facilitate transformation (1). PFK conclude that focusing on joint gains and win–win solutions is possible under conditions of accord but is not possible under conditions of discord. Scholars need to move beyond the “harmony fallacy” (2) because negotiating discord requires splitting costs and benefits, compromising, and partial settlements. However, how then can actors constructively compromise on partial settlements? Sustainability transformations, negotiating discord, and compromising are all “peopled” processes (1). A behavioral negotiation perspective shows how negotiators can navigate conflict constructively by compromising effectively even in contested policy arenas (3).

Psychological research highlights three main barriers to effective compromising: first, negotiators’ exaggerated ambitions (e.g., constituency pressures, power struggles) make them primarily concerned about their own interests and keep negotiators from making concessions (4), even on issues they don’t value much. Second, egocentric perceptions of the other sides’ interests cause negotiators to concede precisely on those issues that the other side may not value (5). Third, negotiators exhibit a self-serving fairness bias: what avoids costs for oneself is perceived as fair to both (6). Together, egoistic motives and biased cognitions cause negotiators to fail to compromise effectively or even to overlook common interests. Consequently, negotiators coerce their counterparts to unilaterally concede or even break up negotiations with an impasse (7) and damaged relationships. Partial settlements, if reached at all, are often watered-down compromises that obscure common ground, do not satisfy the interests of any party (8), and increase losses on all sides (9).

However, the behavioral sciences also provide strategies for negotiators to overcome these psychological barriers toward compromising: actors who are prosocially concerned with the other side’s outcomes develop more trust in the other side, compromise more effectively and end up with fewer impasses (e.g., ref. 10). In addition, negotiators who take the perspective of the other side more accurately judge the counterparts’ actual interests (e.g., refs. 5, and 8), which helps them to compromise on issues the other side values. Also, training negotiators to consider weaknesses in their own fairness judgments immunize them against self-serving biases, making impasses less likely (6). These psychological principles facilitate compromising even when discord seems intractable and only partial settlements might be possible.

A behavioral negotiation perspective identifies the fundamental psychological barriers that emerge under both conditions of accord and under discord. Therefore, understanding how psychological barriers impair compromising and how to improve negotiators’ ability to compromise effectively can help facilitate negotiating discord. Current insights into conflict resolution already address high-conflict settings, such as distributing burdens, negotiating ideological issues, or severe disputes that require third-party intermediation. Future research must apply these insights to sustainability transformations and facilitate much-needed policy action. We hope to deepen the interdisciplinary discourse on promoting policy action.
